# Association of *Calpain10* polymorphisms with polycystic ovarian syndrome susceptibility: a systematic review and meta-analysis with trial sequential analysis

**DOI:** 10.3389/fgene.2023.1153960

**Published:** 2023-09-01

**Authors:** Yamei Li, Ting Han, Yingxia Wang, Jie Gao, Jianglin Zhang, Yinglan Wu, Jiayou Luo

**Affiliations:** ^1^ NHC Key Laboratory for Birth Defect for Research and Prevention, Hunan Provincial Maternal and Child Health Care Hospital, Changsha, China; ^2^ Department of Women Health Care, Hunan Provincial Maternal and Child Healthcare Hospital, Changsha, China; ^3^ Department of Maternal and Child Health, Xiangya School of Public Health, Central South University, Changsha, China

**Keywords:** Calpain10 gene, polymorphism, polycystic ovarian syndrome, systematic review, meta-analysis, false-positive report probability, trial sequential analysis

## Abstract

Insulin resistance plays an important role in the pathogenesis of polycystic ovarian syndrome (PCOS). *Calpain10* (*CAPN10*) gene was the first identified susceptibility gene for type 2 diabetes mellitus and closely related to insulin sensitivity. A lot of research attention has been attracted on the relationship between *CAPN10* polymorphisms and PCOS risk, but they didn’t reach a consistent conclusion. We therefore performed this systematic review and meta-analysis to assess the association of *CAPN10* common variants with PCOS susceptibility. A total of 21 studies were eligible for inclusion. Meta-analyses were done for 5 variants that had at least two data sources: UCSNP-19, -43, −44, −56 and −63. Pooled odds ratios (ORs) and 95% confidence intervals (CIs) were calculated under five genetic models. Subgroup analyses by ethnicity, PCOS diagnostic criteria, and source of controls were conducted. Moreover, false-positive report probability (FPRP) test and trial sequential analysis (TSA) were performed to assess the significant associations. The results showed a possible negative association between UCSNP-19 and PCOS risk (ins/ins vs. del/del + del/ins: OR = 0.84, 95% CI: 0.72–0.98). In subgroup analyses, FPRP test indicated that noteworthy associations were observed in mixed ethnicities for UCSNP-43 (A vs. G: OR = 1.81, 95% CI: 1.17-2.79; AA + AG vs. GG: OR = 2.14, 95% CI: 1.20-3.80) and in Asians for UCSNP-44 (CC vs. TT: OR = 2.07, 95% CI: 1.21-3.51; CC vs. CT + TT: OR = 2.19, 95% CI: 1.31-3.69), but TSA plots showed that the accumulated sample sizes of these associations were insufficient to draw firm conclusions. In summary, our study suggested that UCSNP-19, UCSNP-43, and UCSNP-44 in *CAPN10* gene may be involved in PCOS susceptibility. These findings warrant further studies.

## 1 Introduction

Polycystic ovarian syndrome (PCOS) is the most common reproductive endocrine disorder affecting 5%-20% of women at childbearing age worldwide, depending on the diagnostic criteria (Azziz et al., 2016; Skiba et al., 2018). The condition is characterized by hyperandrogenism, ovulatory dysfunction and polycystic ovarian morphology, and is commonly accompanied by metabolic abnormalities such as insulin resistance, hyperinsulinemia and dyslipidemia (Fauser et al., 2012). PCOS is the primary cause of anovulatory subfertility and increases the risk of pregnancy complications, resulting in major health and economic costs (Roos et al., 2011; Liu et al., 2021). In addition, women with PCOS have increased lifetime risks for type 2 diabetes mellitus (T2DM), cardiovascular events, psychiatric disorders and gynecological cancers (Anagnostis et al., 2018; Cooney and Dokras, 2018). To data, the exact etiology and pathogenesis of PCOS remains unclear. It is believed to result from complex interactions between genetic, behavioral and environmental factors (Azziz et al., 2016). Multiple genes involved in steroid biosynthesis and metabolism, sex hormone regulation, insulin sensitivity and inflammation may be associated with susceptibility to PCOS (Hiam et al., 2019).

*Calpain10* (*CAPN10*) gene is located on the human chromosome 2q37.3 and consists of 15 exons and 14 introns spanning 31 kb. It is the first susceptibility gene for T2DM identified through a genome-wide scan followed by positional cloning (Hanis et al., 1996; Horikawa et al., 2000). *CAPN10* was ubiquitously expressed in most tissues and cell types, and played an important role in insulin secretion and insulin sensitivity (Rasmussen et al., 2002; Sáez et al., 2008). Researchers hypothesized that *CAPN10* gene polymorphisms may contribute to the development of PCOS because that insulin resistance was considered as the common pathologic basis of T2DM and PCOS (Diamanti-Kandarakis and Dunaif, 2012).

Several common polymorphisms in *CAPN10* gene have been studied concerning their potential effect on PCOS susceptibility: UCSNP-19, -22, -43, -44, -56, -58, -63, and -110. Most studies were focusing on UCSNP-19, -43, -44, and -63. However, the results remain controversial. There have been two meta-analyses that examined the association between *CAPN10* gene polymorphisms and PCOS risk in 2012 and 2013, respectively (Huang et al., 2012; Shen et al., 2013). The meta-analysis by Huang et al. (Huang et al., 2012) identified significant associations between UCSNP-19, and -63 and PCOS risk among UCSNP-19, -43, -44 and -63. The latest meta-analysis in 2013 indicated that UCSNP-19, -44 and -63 polymorphisms may be risk factors for PCOS, especially among Asians (Shen et al., 2013). However, the above studies had some obvious shortcomings about study selection, data extraction and statistical analyses (Raihan et al., 2019); besides, adjusted alpha for multiple tests and required information size were not evaluated. Furthermore, several new studies from various countries were conducted to analyze this topic over the past decade. Combining them in updated meta-analysis could increase the overall statistical power to detect an effect.

Therefore, we carried out this systematic review and meta-analysis with trial sequential analysis to pool current evidence together for a more accurate evaluation of the association between *CAPN10* gene polymorphisms and PCOS risk.

## 2 Methods

This systematic review and meta-analysis was designed and reported in accordance with the Preferred Reporting Items for Systematic Reviews and Meta-Analyses guidelines (Supplementary Table S1) (Page et al., 2021).

### 2.1 Search strategy

A comprehensive literature search was conducted through the following electronic databases: The Cochrane Library, Web of Science, PubMed, Embase, Chinese Biomedical Database (CBM), China National Knowledge Infrastructure (CNKI), and Wanfang Database. Keyword search was performed using the following terms: (PCOS OR “polycystic ovary syndrome” OR “polycystic ovarian syndrome”) AND (“calpain 10” OR calpain-10 OR calpain10 OR “CAPN 10” OR CAPN-10 OR CAPN10). The full details of search strategy were listed in Supplementary Appendix SA1. There were no restrictions on either date or language. The search was completed on 4 May 2022.

### 2.2 Inclusion and exclusion criteria

Studies selected in our systematic review had to meet all the following criteria: (1) case-control studies based on unrelated subjects; (2) the diagnosis of PCOS was clear; (3) human studies; (4) the study assessed the association between *CAPN10* polymorphisms and PCOS susceptibility. The exclusion criteria included: (1) case-only studies; (2) family-based studies; (3) comments or review articles; (4) duplicate or overlapping data.

All records obtained through the database search were collected in the EndNote software. After removal of duplicates, all the titles and abstracts were screened to exclude the irrelevant studies and then the full texts of the remaining records were reviewed for eligibility independently by two researchers (YML and TH). Reference lists of the eligible studies were also reviewed.

### 2.3 Data extraction

The following items was extracted from each eligible study: the first author, year of publication, country, ethnicity, sample size, age of cases and controls, diagnostic criteria used for PCOS, source of control subjects, genotyping methods, and allele and genotype frequencies in cases and controls.

### 2.4 Study quality assessment

The quality of included studies was assessed using the Newcastle–Ottawa Scale (NOS) (Stang, 2010). The NOS criteria use a “star” rating system to judge study methodological quality based on three perspectives: selection, comparability, and exposure. Scores were ranged from 0 stars (worst) to 9 stars (best), with equal or higher than 7 indicating that the methodological quality was generally good.

Two researchers (YML and TH) independently performed study screening, data extraction and quality assessment based on the specified selection criteria. All disagreements were resolved by consensus after discussion with the other authors.

### 2.5 Quantitative data synthesis

Meta-analysis was performed for genetic variants with at least two available datasets using the STATA version 14.0 software (Stata Corporation, College Station, TX, United States). Pooled odds ratios (ORs) and 95% confidence intervals (CIs) were calculated under five genetic models: allelic model (mutant [M] allele vs. wild [W] allele), heterozygous model (MW vs. WW), homozygous model (MM vs. WW), recessive model (MM vs. WW + MW), and dominant model (MW + MM vs. WW) (Zintzaras and Lau, 2008).

The Cochrane’s Q test and *I* ^*2*^ index were used to assess the heterogeneity across the eligible studies (Whitehead and Whitehead, 1991; Higgins and Thompson, 2002). *p* value for Q test < 0.1 or *I* ^*2*^ index > 50% indicated significant heterogeneity, and random effect model (DerSimonian–Laird method) was conducted in meta-analysis. Otherwise, the fixed effect model (Mantel–Haenszel method) was applied.

Subgroup analyses were performed by ethnicity, diagnostic criteria, and source of controls to determine the source of heterogeneity. Funnel plots and Egger’s linear regression test were used to assess potential publication bias of included studies (Egger et al., 1997). Sensitivity analyses were conducted by excluding studies whose genotype frequencies in controls exhibited significant deviation from the Hardy–Weinberg equilibrium (HWE). Besides, leave-one-out sensitivity analyses were also performed to evaluate the influence of each study on the overall estimate.

The associations were considered statistically significant if the reported *p* value was <0.05. The false positive report probability (FPRP) values at different prior probability levels for all significant associations were calculated by the Excel spreadsheet which was offered on Wacholder’s website (Wacholder et al., 2004). An FPRP value < 0.2 indicated a noteworthy association.

### 2.6 Trial sequential analysis (TSA)

TSA was conducted to calculate the required information size (RIS), and to assess if quantitative findings were robust (Wetterslev et al., 2008). We estimated the RIS based on a type I error of 5%, power of 80% and relative risk reduction assumption of 20% (Møller et al., 2008). If a trial sequential monitoring boundary is crossed before the RIS is reached in a cumulative meta-analysis, firm evidence may have been established and further studies were superfluous; if the boundaries are not surpassed, it is most probably necessary to conduct more studies in order to detect or reject a certain association (Møller et al., 2008). TSA was performed using TSA 0.9.5.10 Beta software (Copenhagen Trial Unit, Denmark).

## 3 Results

### 3.1 Study selection

**FIGURE 1:**
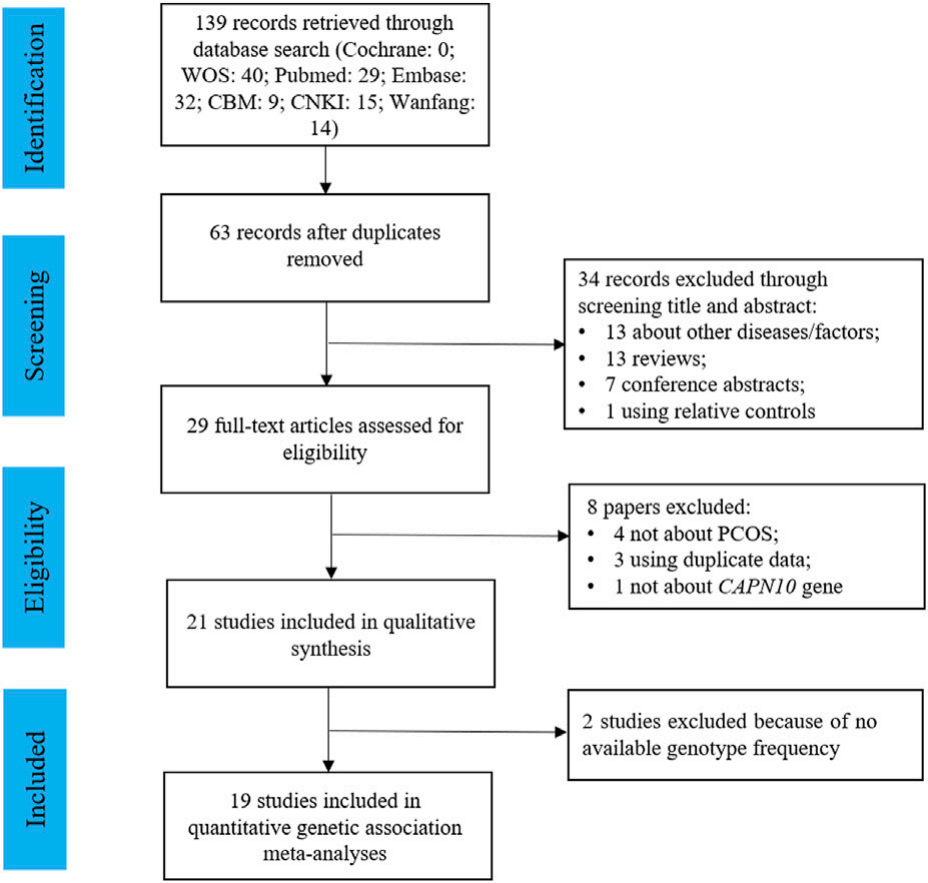
Flowchart of the study selection.

Figure 1 presented the study selection process for this review. Initially, a total of 139 articles were identified by searching various databases, and 63 non-duplicated articles were included in the title and abstract screening. 34 articles were excluded for not about PCOS (*n* = 7), not about *CAPN10* polymorphisms (*n* = 6), reviews (*n* = 13), conference abstracts (*n* = 7), or family-based studies (*n* = 1), and the remaining 29 papers were included in the full-text review. Finally, 21 studies were eligible for the systematic review, and 19 of them had sufficient data for meta-analysis for 5 common variants: UCSNP-19, -43, -44, -56 and -63.

### 3.2 Characteristics of eligible studies

Table 1 summarized the main characteristics of the 21 studies included in the systematic review. All the studies were published between 2002 and 2017. Among them, 9 studies were conducted in Asians, 8 studies in Caucasians, 3 studies in mixed population, and 1 in Africans. 11 studies used population-based controls, and 10 studies used hospital-based controls. The majority of studies adopted the revised Rotterdam criteria for definition of PCOS (*n* = 19), and the remaining 2 studies adopted the National Institutes of Health (NIH) criteria. All the included studies had good quality with the NOS score ranging from 7 to 9.

**TABLE 1:** Basic characteristics of all eligible studies in systematic review.

First author	Year	Country	Ethnicity	Diagnostic criteria	Sample size		Source of controls	Genotype method	Studied variants*	NOS score
					Cases	Controls				
Gonzalez et al. (2002)	2002	Spain	Caucasian	PCO + HA + OA fulfilled NIH 1990	55	93	PB	real-time PCR	UCSNP-19/43/**44**/63	8
Haddad et al. (2002)	2002	UK	Caucasian	PCO + HA ± OA fulfilled Rotterdam 2003	185	525	PB	UCSNP-19: PCR,	UCSNP-19/43/44/63	8
								UCSNP-43/44: MS-PCR, UCSNP-63: PCR-RFLP		
Haap et al. (2005)	2005	Germany	Caucasian	Rotterdam 2003	57	563	PB	Direct sequencing	UCSNP-43/44/45	8
Sun et al. (2007a)	2007	China	Asian	Rotterdam 2003	107	126	HB	PCR-RFLP	UCSNP-43	8
Sun et al. (2007b)	2007	China	Asian	Rotterdam 2003	98	111	HB	PCR-RFLP	UCSNP-19	8
Wiltgen et al. (2007)	2007	Brazil	Mixed	OA + HA ± PCO fulfilled Rotterdam 2003	59	29	HB	UCSNP-19: PCR,	UCSNP-19/43/63	7
								UCSNP-43: MS-PCR, UCSNP-63: PCR-RFLP		
Vollmert et al. (2007)	2007	Germany	Caucasian	Rotterdam 2003	146	606	PB	MALDI-TOF MS	UCSNP-**19**/22/43/44/**56**/58/63/110	8
Diao et al. (2008)	2008	China	Asian	Rotterdam 2003	334	304	HB	PCR Tm-shift	UCSNP-56	8
Lee et al. (2008)	2008	Korea	Asian	Rotterdam 2003	164	352	PB	UCSNP-19: PCR,	UCSNP-19/43/63	9
								UCSNP-43: PCR-RFLP, UCSNP-63: Direct sequencing		
Márquez et al. (2008)	2008	Chile	Mixed	NIH 1990	50	70	HB	UCSNP-19: PCR,	UCSNP-19/**43**/63	8
								UCSNP-43/63: PCR-RFLP		
Unsal et al. (2009)	2009	Turkey	Caucasian	Rotterdam 2003	44	50	HB	PCR-RFLP	UCSNP-19/43/44/63	8
Yilmaz et al. (2009)	2009	Turkey	Caucasian	Rotterdam 2003	107	114	HB	PCR-RFLP	UCSNP-19/43/**44**/63	8
Li et al. (2010)	2010	China	Asian	Rotterdam 2003	96	96	HB	PCR-RFLP	UCSNP-43	8
Dasgupta et al. (2012)	2012	India	Asian	Rotterdam 2003	250	299	PB	Direct sequencing	UCSNP-19/43/**44**/56/63	9
Ben Salem et al. (2014)	2014	Tunisia	African	Rotterdam 2003	127	150	HB	UCSNP-19: PCR,	UCSNP-19/43/63	7
								UCSNP-43/63: PCR-RFLP		
Anastasia et al. (2015)	2015	Greece	Caucasian	Rotterdam 2003	668	200	PB	MALDI-TOF MS	UCSNP-43	8
Deng et al. (2015)	2015	China	Asian	Rotterdam 2003	107	111	PB	PCR-RFLP	UCSNP-**19**	9
Flores-Martínez et al. (2015)	2015	Mexico	Mixed	Rotterdam 2003	55	46	PB	UCSNP-19: PCR,	UCSNP-19/63	8
								UCSNP-63: PCR-RFLP		
Khazamipour et al. (2015)	2015	Iran	Caucasian	Rotterdam 2003	90	90	PB	PCR-RFLP	UCSNP-44	8
Reddy et al. (2016)	2016	India	Asian	Rotterdam 2003	248	210	PB	Sequenom MassARRAY	UCSNP-43/63	8
Thangavelu et al. (2017)	2017	India	Asian	Rotterdam 2003	169	169	HB	real-time PCR	UCSNP-58/110	8

PCO, polycystic ovaries under B-ultrasound; HA, hyperandrogenism; OA, oligomenorrhea or amenorrhea; PB, population-based; HB, hospital-based; PCR, polymerase chain reaction; MS-PCR, Mutagenically separated PCR; PCR-RFLP, PCR-restriction fragment length polymorphism; PCR, Tm-shift; PCR, melting temperature shift; MALDI-TOF MS, matrix-assisted laser desorption/ionization time-of-flight mass spectroscopy. *The variants in bold were significantly associated with PCOS.

Nine common variants in *CAPN10* gene were investigated, including UCSNP-19 (rs3842570 Del>Ins), UCSNP-22 (rs2953152 G>A), UCSNP-43 (rs3792267 G>A), UCSNP-44 (rs2975760 T>C), UCSNP-45 (rs2241766 A>C), UCSNP-56 (rs2975762 G>A), UCSNP-58 (rs2975766 A>G), UCSNP-63 (rs5030952 C>T) and UCSNP-110 (rs7607759 A>G). UCSNP-43 was the focus of most studies (*n* = 15), followed by UCSNP-19 (*n* = 13), UCSNP-63 (*n* = 12) and UCSNP-44 (*n* = 8). There were 3, 2 and 2 studies for UCSNP-56, UCSNP-58 and UCSNP-110, and one study for each of UCSNP-22 and UCSNP-45.

As shown in Supplementary Table S2, more than two available sets of allele or genotype frequency data were extracted from these studies about UCSNP-19, -43, -44, -56, and -63. The genotype distribution in controls was consistent with HWE for UCSNP-19, and -56. However, significant deviation from HWE was detected in genotype frequencies of controls in one study for UCSNP-43 (Dasgupta et al., 2012), in two studies for UCSNP-44 (Yilmaz et al., 2009; Dasgupta et al., 2012) and in three studies for UCSNP-63 (Lee et al., 2008; Yilmaz et al., 2009; Dasgupta et al., 2012).

### 3.3 Association between UCSNP-19 and PCOS

**FIGURE 2:**
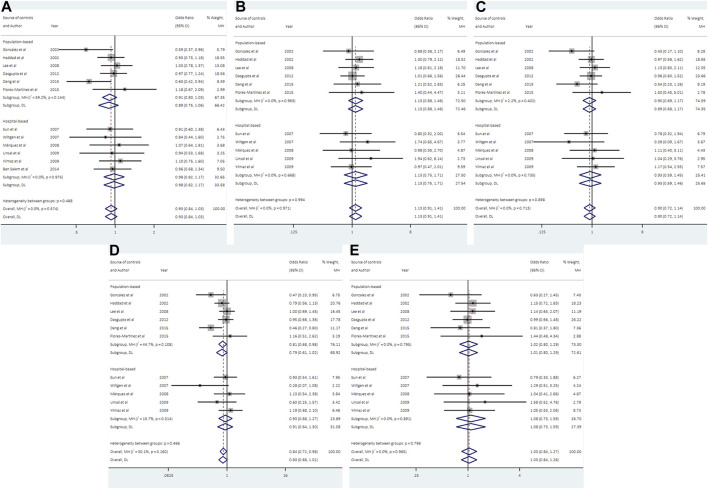
Forest plots of association between UCSNP-19 and PCOS susceptibility by source of controls [**(A)**: allelic model, **(B)** heterozygous model, **(C)** homozygous model, **(D)** recessive model, **(E)** dominant model].

12 studies consisting of 1299 PCOS patients and 1948 controls were included in the meta-analysis for UCSNP-19. The pooled estimate of recessive model revealed a significant association (ins/ins vs. del/del + del/ins: OR = 0.84, 95% CI: 0.72–0.98) (Table 2; Figure 2). In the subgroup analyses, only the recessive model in studies with population-based controls showed a significant association (ins/ins vs. del/del + del/ins: OR = 0.81, 95% CI: 0.68–0.98) (Table 3; Figure 2). Further, the significant association between UCSNP-19 and PCOS remained noteworthy at the prior probability of 0.1 and FPRP threshold of 0.2 (Supplementary Table S3).

**TABLE 2:** Overall meta-analysis results for the association between *CAPN10* polymorphisms and PCOS risk.

Variant	Genetic models	n	Cases	Controls	*P*_heterogeneity_	*I* ^*2*^(*%*)	Statistical method	Pooled OR (95% CI)	*P*	Egger’s *t* value	*P*
UCSNP-19											
	Allelic model	12	1299	1948	0.574	0	Fixed	0.93 (0.84, 1.03)	0.176	−0.62	0.549
	Heterozygous model	11	1172	1798	0.971	0	Fixed	1.13 (0.91, 1.41)	0.260	0.69	0.507
	Homozygous model	11	1172	1798	0.713	0	Fixed	0.90 (0.72, 1.14)	0.387	−0.96	0.363
	Recessive model	11	1172	1798	0.160	30.1	Fixed	0.84 (0.72, 0.98)	0.031	−1.20	0.262
	Dominant model	11	1172	1798	0.965	0	Fixed	1.03 (0.84, 1.27)	0.759	0.17	0.872
UCSNP-43											
	Allelic model	13	2124	2607	0.126	32.1	Fixed	0.99 (0.87, 1.12)	0.841	0.52	0.611
	Heterozygous model	12	1997	2457	0.230	21.7	Fixed	1.03 (0.88, 1.21)	0.705	0.04	0.966
	Homozygous model	10	1726	1991	0.157	31.4	Fixed	1.06 (0.73, 1.52)	0.772	0.82	0.436
	Recessive model	10	1726	1991	0.207	25.8	Fixed	1.01 (0.71, 1.44)	0.962	0.66	0.527
	Dominant model	12	1997	2457	0.200	24.8	Fixed	1.02 (0.88, 1.19)	0.786	0.57	0.581
UCSNP-44											
	Allelic model	6	692	1613	0.040	57.0	Random	1.21 (0.92, 1.61)	0.179	−0.23	0.832
	Heterozygous model	6	692	1613	0.014	64.9	Random	1.21 (0.82, 1.79)	0.344	0.39	0.718
	Homozygous model	5	585	1499	0.091	50.2	Random	1.11 (0.41, 2.97)	0.835	−1.77	0.176
	Recessive model	5	585	1499	0.083	51.4	Random	1.09 (0.40, 2.93)	0.868	−2.10	0.127
	Dominant model	6	692	1613	0.031	59.4	Random	1.26 (0.89, 1.78)	0.191	0.33	0.755
UCSNP-56											
	Allelic model	2	582	602	0.386	0	Fixed	0.92 (0.78, 1.08)	0.304	-	-
	Heterozygous model	2	582	602	0.570	0	Fixed	0.92 (0.71, 1.18)	0.501	-	-
	Homozygous model	2	582	602	0.251	24.0	Fixed	0.88 (0.62, 1.24)	0.459	-	-
	Recessive model	2	582	602	0.130	56.3	Random	0.91 (0.57, 1.45)	0.688	-	-
	Dominant model	2	582	602	0.966	0	Fixed	0.90 (0.71, 1.15)	0.404	-	-
UCSNP-63											
	Allelic model	11	1329	1926	0.911	0	Fixed	1.02 (0.87, 1.20)	0.785	−1.38	0.200
	Heterozygous model	10	1202	1776	0.843	0	Fixed	1.05 (0.83, 1.33)	0.684	−0.86	0.416
	Homozygous model	9	1158	1726	0.826	0	Fixed	1.02 (0.73, 1.44)	0.889	−0.88	0.410
	Recessive model	9	1158	1726	0.798	0	Fixed	1.01 (0.72, 1.42)	0.932	−0.94	0.376
	Dominant model	10	1202	1776	0.890	0	Fixed	1.04 (0.86, 1.27)	0.669	−1.04	0.328

**TABLE 3:** Stratified meta-analysis results for the association between *CAPN10* polymorphisms and PCOS risk.

Variant/subgroup	Allelic model		Heterozygous model		Homozygous model		Recessive model		Dominant model	
	OR (95% CI)	*P*	OR (95% CI)	*P*	OR (95% CI)	*P*	OR (95% CI)	*P*	OR (95% CI)	*P*
UCSNP-19										
Caucasian	0.91 (0.76, 1.09)	0.290	1.19 (0.84, 1.69)	0.332	0.90 (0.62, 1.30)	0.559	0.79 (0.61, 1.03)	0.076	1.05 (0.76, 1.47)	0.764
Asian	0.92 (0.79, 1.07)	0.281	1.04 (0.77, 1.42)	0.786	0.90 (0.65, 1.23)	0.509	0.86 (0.70, 1.07)	0.172	0.97 (0.73, 1.30)	0.853
Mixed	1.04 (0.75, 1.45)	0.810	1.33 (0.73, 2.44)	0.350	0.97 (0.49, 1.91)	0.920	0.94 (0.57, 1.55)	0.793	1.23 (0.70, 2.16)	0.480
PB	0.91 (0.80, 1.03)	0.132	1.13 (0.88, 1.46)	0.340	0.90 (0.69, 1.17)	0.418	0.81 (0.68, 0.98)	0.026	1.02 (0.80, 1.29)	0.899
HB	0.98 (0.82, 1.17)	0.828	1.13 (0.75, 1.71)	0.550	0.93 (0.59, 1.45)	0.743	0.93 (0.68, 1.27)	0.651	1.08 (0.73, 1.59)	0.703
NIH	0.78 (0.55, 1.11)	0.170	0.92 (0.47, 1.81)	0.817	0.67 (0.34, 1.32)	0.247	0.71 (0.43, 1.18)	0.181	0.79 (0.42, 1.47)	0.450
Rotterdam	0.95 (0.85, 1.06)	0.321	1.16 (0.92, 1.46)	0.205	0.94 (0.74, 1.20)	0.610	0.86 (0.73, 1.01)	0.067	1.07 (0.86, 1.32)	0.560
UCSNP-43										
Caucasian	1.03 (0.87, 1.23)	0.716	1.02 (0.82, 1.27)	0.832	1.09 (0.70, 1.71)	0.704	1.08 (0.69, 1.68)	0.739	1.03 (0.84, 1.27)	0.775
Asian	0.85 (0.68, 1.06)	0.147	0.93 (0.72, 1.19)	0.554	0.49 (0.22, 1.11)	0.089	0.48 (0.21, 1.09)	0.078	0.88 (0.69, 1.12)	0.303
Mixed	1.81 (1.17, 2.79)	0.007	1.95 (1.08, 3.54)	0.028	3.53 (1.15, 10.86)	0.028	2.40 (0.84, 6.86)	0.102	2.14 (1.20, 3.80)	0.010
PB	0.98 (0.85, 1.13)	0.764	1.03 (0.86, 1.23)	0.788	0.87 (0.58, 1.30)	0.506	0.86 (0.58, 1.28)	0.458	1.00 (0.84, 1.18)	0.977
HB	1.01 (0.80, 1.29)	0.915	1.05 (0.75, 1.48)	0.761	2.81 (1.09, 7.24)	0.033	2.19 (0.89, 5.40)	0.090	1.12 (0.80, 1.56)	0.514
NIH	1.44 (0.98, 2.11)	0.062	1.58 (0.94, 2.67)	0.086	1.84 (0.76, 4.46)	0.180	1.47 (0.62, 3.46)	0.383	1.61 (0.98, 2.64)	0.060
Rotterdam	0.95 (0.83, 1.08)	0.399	0.99 (0.84, 1.17)	0.881	0.95 (0.64, 1.41)	0.790	0.93 (0.63, 1.39)	0.734	0.97 (0.83, 1.14)	0.743
UCSNP-44										
Caucasian	1.17 (0.80, 1.72)	0.415	1.35 (0.87, 2.09)	0.177	0.71 (0.18, 2.82)	0.625	0.69 (0.19, 2.45)	0.561	1.31 (0.82, 2.09)	0.261
Asian	1.34 (1.02, 1.77)	0.036	0.80 (0.53, 1.21)	0.288	2.07 (1.21, 3.51)	0.007	2.19 (1.31, 3.69)	0.003	1.11 (0.78, 1.57)	0.563
PB	1.21 (0.86, 1.71)	0.281	1.10 (0.73, 1.66)	0.645	1.21 (0.42, 3.48)	0.730	1.17 (0.40, 3.41)	0.775	1.17 (0.84, 1.65)	0.354
HB	1.14 (0.52, 2.51)	0.747	1.39 (0.48, 4.00)	0.545	0.34 (0.01, 8.73)	0.517	0.37 (0.02, 9.34)	0.547	1.34 (0.43, 4.11)	0.613
NIH	2.31 (1.22, 4.37)	0.010	2.50 (1.12, 5.59)	0.025	2.94 (0.62, 13.87)	0.173	2.35 (0.51, 10.93)	0.275	2.57 (1.22, 5.44)	0.013
Rotterdam	1.13 (0.87, 1.46)	0.372	1.09 (0.74, 1.61)	0.666	0.74 (0.19, 2.82)	0.654	0.74 (0.18, 3.00)	0.678	1.14 (0.83, 1.58)	0.419
UCSNP-63										
Caucasian	1.07 (0.80, 1.44)	0.640	1.07 (0.77, 1.49)	0.697	1.15 (0.45, 2.96)	0.768	1.11 (0.44, 2.84)	0.821	1.07 (0.78, 1.48)	0.662
Asian	1.03 (0.82, 1.29)	0.828	0.92 (0.61, 1.41)	0.716	1.07 (0.73, 1.56)	0.735	1.07 (0.74, 1.56)	0.726	1.00 (0.75, 1.34)	0.996
Mixed	0.99 (0.64, 1.52)	0.954	1.22 (0.72, 2.05)	0.458	0.48 (0.12, 1.98)	0.311	0.44 (0.11, 1.78)	0.248	1.10 (0.67, 1.82)	0.697
PB	1.05 (0.87, 1.27)	0.610	1.03 (0.78, 1.36)	0.855	1.07 (0.74, 1.55)	0.712	1.07 (0.74, 1.55)	0.705	1.05 (0.83, 1.31)	0.706
HB	0.96 (0.72, 1.28)	0.783	1.11 (0.72, 1.71)	0.641	0.81 (0.35, 1.90)	0.626	0.76 (0.33, 1.76)	0.519	1.04 (0.70, 1.56)	0.840
NIH	1.12 (0.78, 1.60)	0.535	1.28 (0.86, 1.90)	0.223	0.48 (0.09, 2.52)	0.387	0.43 (0.08, 2.22)	0.313	1.21 (0.82, 1.79)	0.331
Rotterdam	1.00 (0.84, 1.19)	0.997	0.95 (0.71, 1.27)	0.717	1.07 (0.75, 1.51)	0.721	1.06 (0.75, 1.50)	0.733	0.99 (0.79, 1.25)	0.949

### 3.4 Association between UCSNP-43 and PCOS

**FIGURE 3:**
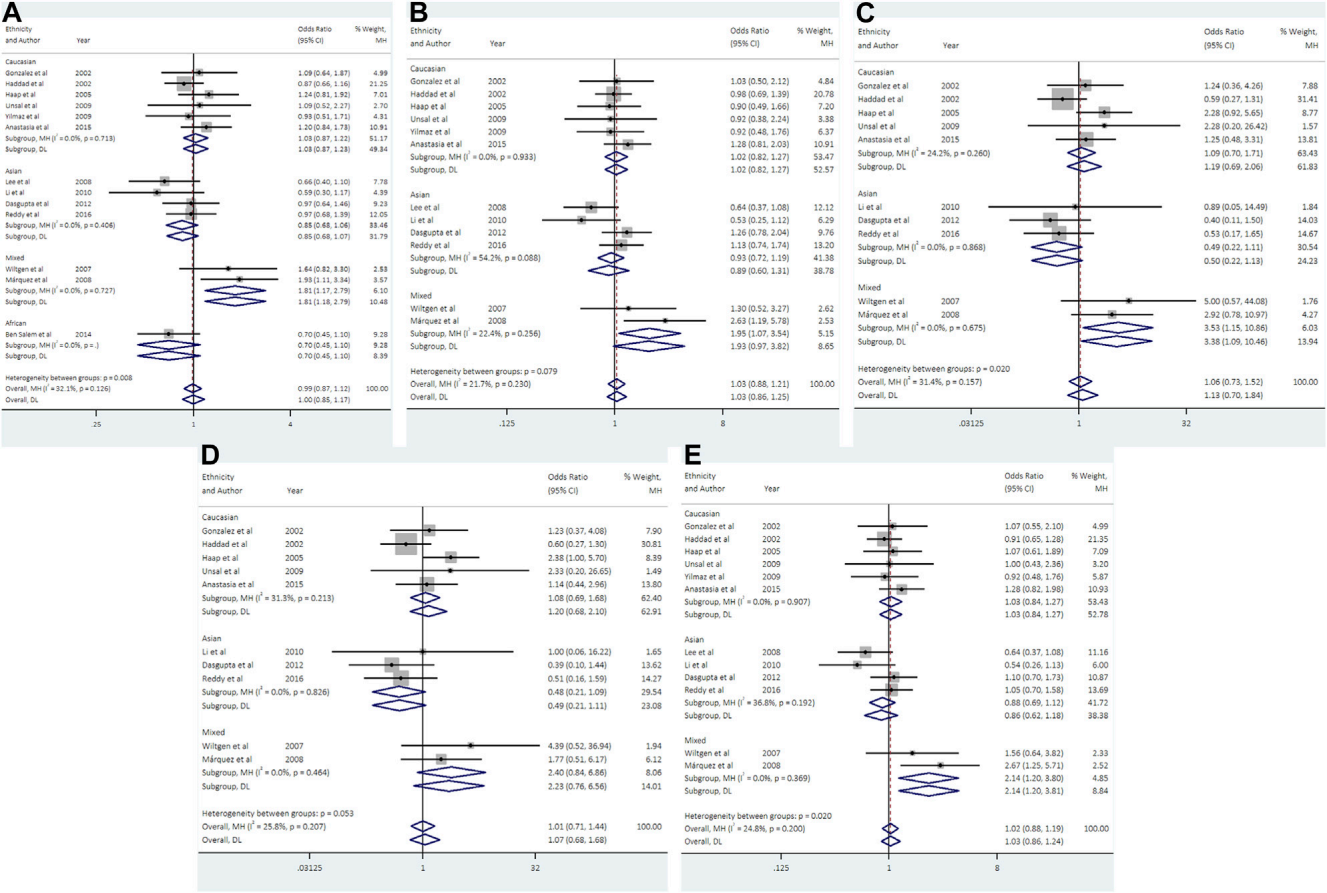
Forest plots of association between UCSNP-43 and PCOS susceptibility by ethnicity [**(A)**: allelic model, **(B)** heterozygous model, **(C)** homozygous model, **(D)** recessive model, **(E)** dominant model].

With regard to UCSNP-43, 13 studies involving 2124 cases and 2607 controls met the eligibility criteria for meta-analysis. No significant associations between UCSNP-43 and PCOS susceptibility were observed under all genetic models (Table 2; Figure 3). In the subgroup analyses by ethnicity, significant associations were observed in allelic model (A vs. G: OR = 1.81, 95% CI: 1.17-2.79), heterozygous model (AG vs. GG: OR = 1.95, 95% CI: 1.08-3.54), homozygous model (AA vs. GG: OR = 3.53, 95% CI: 1.15-10.86) and dominant model (AA + AG vs. GG: OR = 2.14, 95% CI: 1.20-3.80) in mixed ethnicities (Table 3; Figure 3). When stratified by source of controls, the significant association of homozygous model (AA vs. GG: OR = 2.81, 95% CI: 1.09-7.24) was found in the subgroup of hospital-based controls (Table 3). But FPRP test indicated that the associations were noteworthy in allelic model and dominant model among mixed ethnicities (Supplementary Table S2).

### 3.5 Association between UCSNP-44 and PCOS

**FIGURE 4:**
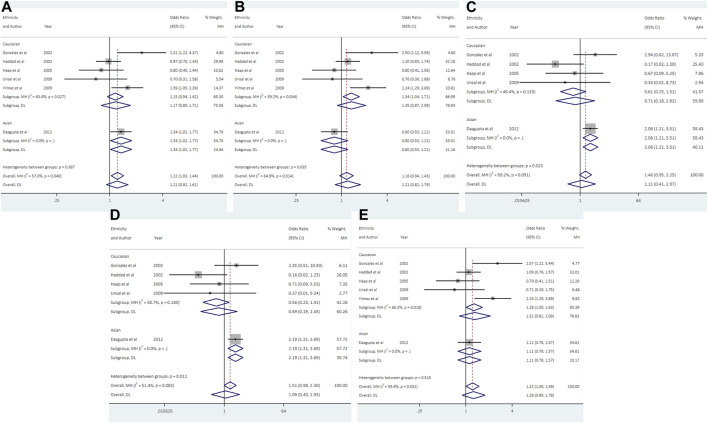
Forest plots of association between UCSNP-44 and PCOS susceptibility by ethnicity [**(A)**: allelic model, **(B)** heterozygous model, **(C)** homozygous model, **(D)** recessive model, **(E)** dominant model].

Data from 6 studies (692 cases and 1613 controls) showed that UCSNP-44 was not significantly associated with PCOS risk in all genetic models (Table 2; Figure 4). In the subgroup analyses by ethnicity, significant associations were observed in allelic model (C vs. T: OR = 1.34, 95% CI: 1.02-1.77), homozygous model (CC vs. TT: OR = 2.07, 95% CI: 1.21-3.51) and recessive model (CC vs. CT + TT: OR = 2.19, 95% CI: 1.31-3.69) in Asians (Table 3; Figure 4). When stratified according to PCOS diagnostic criteria, we observed significant associations of allelic model (C vs. T: OR = 2.31, 95% CI: 1.22-4.37), heterozygous model (CT vs. TT: OR = 2.50, 95% CI: 1.12-5.59) and dominant model (CC + CT vs. TT: OR = 2.57, 95% CI: 1.22-5.44) in NIH subgroup (Table 3). The significant associations remained noteworthy in homozygous model and recessive model among Asians in the FPRP tests (Supplementary Table S2).

### 3.6 Association between UCSNP-56, UCSNP-63 and PCOS

For UCSNP-56, 2 studies consisting of 582 cases and 602 controls were included in the meta-analysis. No significant associations were observed under all genetic models (Table 2; Supplementary Figure S1). The subgroup analyses were not performed because of the limited studies included.

For UCSNP-63, 11 studies involving 1329 cases and 1926 controls were eligible for meta-analysis. No significant associations were observed under all genetic models in overall and subgroup analyses (Tables 2, 3; Supplementary Figure S2).

### 3.7 Publication bias

The results of Egger’s test still did not suggest any evidence of publication bias in the analysis (Table 2). Besides, the shape of the funnel plots did not reveal any evidence of obvious asymmetry in all genetic models for these five SNPs (Supplementary Figures S3–S7).

### 3.8 Sensitivity analyses

Sensitivity analyses were carried out by excluding studies not in HWE for UCSNP-43, UCSNP-44, and UCSNP-63, and all the results remained practically unchanged (Supplementary Table S3). Sensitivity analyses were also performed to evaluate the influence of a specific study on the overall estimate. We observed that omission of any single study had little effect on the combined results for UCSNP-43 and UCSNP-63. For UCSNP-19, the pooled ORs were insignificant under the recessive model when omitting each of the following studies: Gonzalez et al., 2002, Haddad et al., 2002, Wiltgen et al., 2007, and Deng et al., 2015. Conversely, the pooled ORs of homozygous model and recessive model for UCSNP-44 become statistically significant after exclusion of the study by Haddad et al., 2002 (Supplementary Table S4).

### 3.9 Trial sequential analysis

**FIGURE 5:**
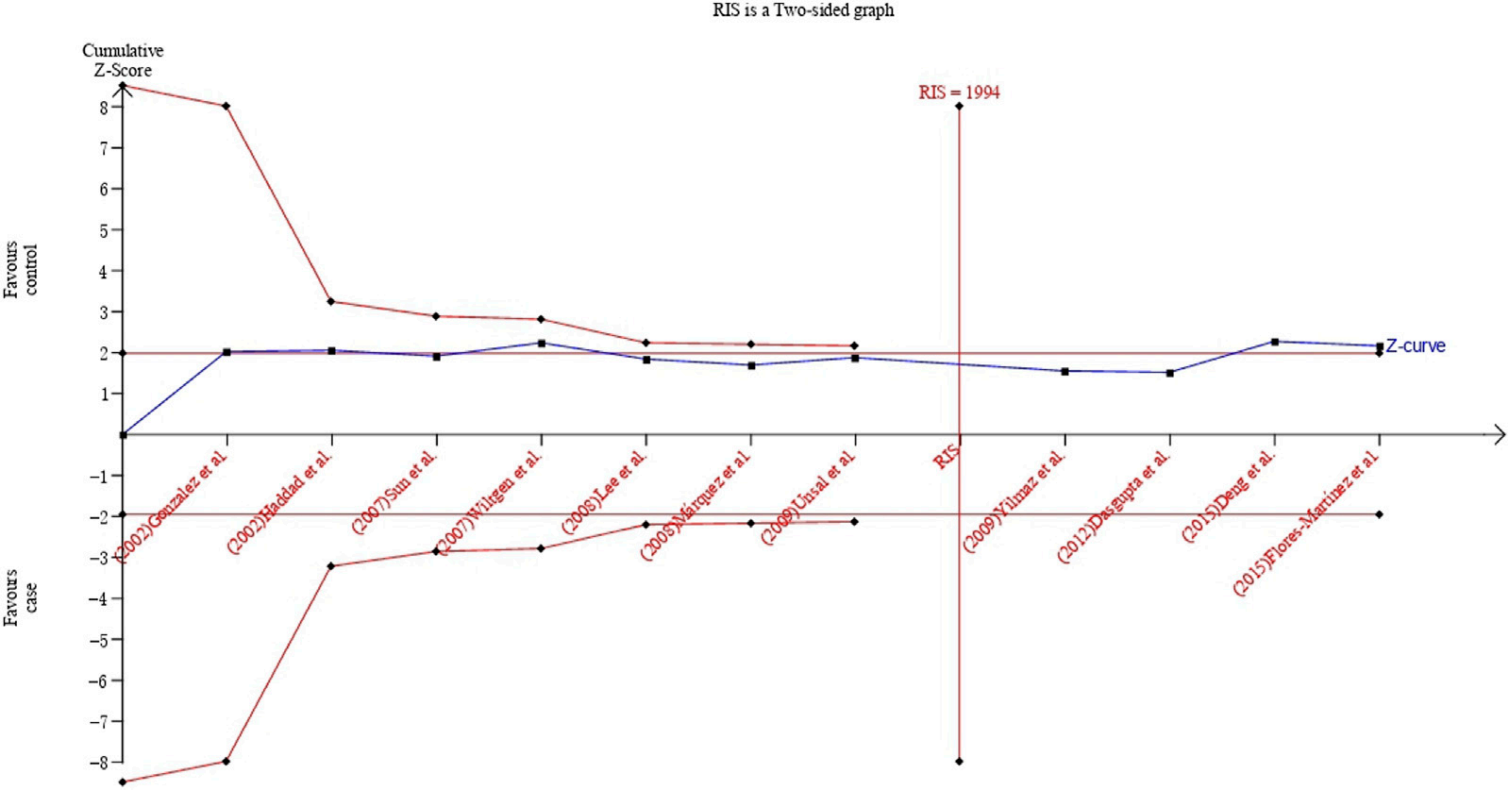
TSA plot of the recessive model for the association between UCSNP-19 and PCOS susceptibility.

The TSA showed that cumulative Z curve reached the RIS and crossed the conventional boundary, but it did not cross the trial sequential monitoring boundary, suggesting that the correlation within UCSNP-19 polymorphism and PCOS susceptibility may be invalid and more studies are needed for stable conclusion (Figure 5). For UCSNP-43 and UCSNP-44, the cumulative Z curves crossed the conventional boundary, but it did not cross the trial sequential monitoring boundary and reach the RIS, which suggested that more studies were needed to confirm these findings in subgroup analyses (Supplementary Figures S8–S11).

## 4 Discussion

The identification of genetic variants associated with PCOS and related traits has the potential to inform our understanding for pathogenesis of PCOS. The current study provided a systematic assessment of the association between *CAPN10* polymorphisms and susceptibility to PCOS. The results showed a possible negative association between UCSNP-19 and PCOS susceptibility. UCSNP-43 and UCSNP-44 may be associated with increased risk of PCOS among mixed ethnicities and Asians, respectively. However, no significant association was observed between UCSNP-22, -45, -56, -58, -63 and -110 polymorphisms and PCOS risk.

Our finding about the significant association of UCSNP-19 with PCOS was consistent with the previous meta-analyses by Huang et al. (2012) and Shen et al. (2013). UCSNP-19, localized in intron 6 of *CAPN10* gene, is a 2-allele polymorphism consisting of 2 or 3 copies of 32-bp sequence. The association may be attributed to the effect of UCSNP-19 polymorphism or the possible haplotypes of *CAPN10* gene. Although UCSNP-19 did not alter the amino acid sequence, the variable number of tandem repeat polymorphism may be involved in the transcription modification and consequent protein expression (Nakamura et al., 1998; Vollmert et al., 2007). The haplotype combination defined by UCSNP-43, -19, and -63 alleles was reported to confer increased risk of PCOS or T2DM or insulin resistance in different studies (Horikawa et al., 2000; Ehrmann et al., 2002; Sáez et al., 2008; Lee et al., 2009). Besides, UCSNP-19 and UCSNP-56 were found to be in strong linkage disequilibrium and showed significant association with PCOS in German women (Vollmert et al., 2007).

In subgroup analyses, ethnic-specific genetic associations of UCSNP-43 and -44 with PCOS were observed in our study. The role of these two SNPs in PCOS susceptibility have been attracted much attention due to the strong relevance between two SNPs and T2DM. The UCSNP-43 in intron 3 was the variant most strongly linked with T2DM among Mexican Americans (Horikawa et al., 2000). Afterwards, the UCSNP-43, either individually or in combination with UCSNP-19 and -63, was found to contribute to PCOS susceptibility in specific population (Ehrmann et al., 2002; Márquez et al., 2008; Lee et al., 2009). UCSNP-44, located in a transcription enhancer element next to UCSNP-43, was shown to be associated with T2DM in Mexican Americans and Europeans (Evans et al., 2001) and related to PCOS in several studies (Gonzalez et al., 2002; Yilmaz et al., 2009; Dasgupta et al., 2012). Moreover, experimental studies have suggested that the polymorphisms defined by UCSNP-43 and UCSNP-44 in intron 3 appear to regulate the expression of *CAPN10* gene (Horikawa et al., 2000; Evans et al., 2001). Ethnicity is considered as a multidimensional construct that may vary greatly in genetic background, socioeconomic status, and lifestyle (Beatty Moody et al., 2021). These factors might have complex influence on effect allele frequencies of susceptibility variants, gene-environment interaction, and the incidence of PCOS (Kim and Choi, 2019; Khil et al., 2022). But it is worth noting that some ethnic subgroups in our study contained few studies without sufficient statistical power, and more studies in various populations are needed to confirm the results.

However, the present study did not show significant association between UCSNP-63 and PCOS susceptibility overall or within any subgroup, which was somewhat different from the previous meta-analyses (Huang et al., 2012; Shen et al., 2013). The discrepancies may be explained by the improper data extraction in the previous studies. The dataset erroneously derived from the haplotype data of a Korean study was found to be the outlier of forest plots for UCSNP-63, which were also proved by the leave-one-out sensitivity analyses (Huang et al., 2012; Shen et al., 2013). In fact, the association between UCSNP-63 and PCOS had not been observed in the individual studies. Our study further confirmed that the single variant UCSNP-63 probably has no effect on PCOS in the pooled sample.

Although the mechanisms underlying PCOS remain elusive, recent studies have suggested that hyperandrogenism and insulin resistance were the core etiology and primary endocrine characteristics of this condition (Diamanti-Kandarakis and Dunaif, 2012; Wang et al., 2019). Insulin resistance was found in approximately three-quarters of affected women, and played a crucial role in the pathophysiology of PCOS (Tosi et al., 2017; Ding et al., 2021). Hyperinsulinemia from insulin resistance contributes to hyperandrogenism via stimulation of ovarian androgen secretion and inhibition of hepatic sex hormone-binding globulin synthesis, leading to follicle arrest and anovulation (Diamanti-Kandarakis and Dunaif, 2012). In turn, androgen excess could impair insulin action in skeletal muscle and adipose tissue of women with PCOS, potentially setting up a vicious cycle (Corbould, 2008). As in T2DM, intrinsic defects in insulin secretion and action are critical for PCOS development (Diamanti-Kandarakis and Papavassiliou, 2006; Tripathy and Chavez, 2010; Shaaban et al., 2021). *CAPN10* gene is the first putative diabetes gene and encodes the cysteine protease calpain10 that involved in pro-insulin processing and insulin secretion and action (Sreenan et al., 2001). Multiple polymorphisms in *CAPN10* gene individually or in combination were demonstrated to regulate gene expression and be associated with insulin resistance and PCOS (Horikawa et al., 2000; Shaaban et al., 2021). Accordingly, *CAPN10* may be an important susceptibility gene for PCOS.

### 4.1 Strengths and limitations

This systematic review had several strengths. First, our study has been well-designed with explicit criteria and methods for study selection, data extraction and data analysis compared with the previous meta-analyses (Huang et al., 2012; Shen et al., 2013), which make it advantageous to provide powerful and valid results. Second, FPRP test and TSA were used to justify the results and enhance the reliability of the results. However, our findings should be interpreted in the light of the following limitations. First of all, only published literatures with sufficient data were included in the meta-analysis. Therefore, publication bias may have occurred, even though the results of Egger’s linear regression tests did not show it. Second, the meta-analyses for some variants (e.g., UCSNP-56, UCSNP-44) with few studies and limited sample size may not have enough statistical power to explore the real association. Further large-scale validation studies are needed for these polymorphisms. Third, significant changes were observed in the leave-one-out sensitivity analyses of the recessive model for UCSNP-19 as well as the homozygous and recessive model for UCSNP-44, which had some impact on the robustness of the conclusions. Finally, PCOS is thought to be a complex disease resulting from interactions between multiple genes and environmental factors, but it is difficult to eliminate the effect of confounding factors in meta-analysis, which may affect the final results.

In conclusion, our study showed a possible negative association between UCSNP-19 and PCOS susceptibility, while UCSNP-43 and UCSNP-44 may be associated with increased risk of PCOS in specific ethnicities. Although the findings reflected the correlation between *CAPN10* gene polymorphisms and PCOS, they were not robust enough to withstand statistical interrogation. Further rigorous studies in various populations are needed to draw more definite conclusions.

## Data Availability

The original contributions presented in the study are included in the article/Supplementary Material, further inquiries can be directed to the corresponding authors.

## Supplementary material

SUPPLEMENTARY FIGURE S1 Forest plots of association between UCSNP-56 and PCOS susceptibility [**(A)**: allelic model, **(B)**: heterozygous model, **(C)**: homozygous model, **(D)**: recessive model, **(E)**: dominant model].

SUPPLEMENTARY FIGURE S2 Forest plots of association between UCSNP-63 and PCOS susceptibility [**(A)**: allelic model, **(B)**: heterozygous model, **(C)**: homozygous model, **(D)**: recessive model, **(E)**: dominant model].

SUPPLEMENTARY FIGURE S3 Funnel plots of the five genetic models for the association between UCSNP-19 and PCOS susceptibility [**(A)**: allelic model, **(B)**: heterozygous model, **(C)**: homozygous model, **(D)**: recessive model, **(E)**: dominant model].

SUPPLEMENTARY FIGURE S4 Funnel plots of the five genetic models for the association between UCSNP-43 and PCOS susceptibility [**(A)**: allelic model, **(B)**: heterozygous model, **(C)**: homozygous model, **(D)**: recessive model, **(E)**: dominant model].

SUPPLEMENTARY FIGURE S5 Funnel plots of the five genetic models for the association between UCSNP-44 and PCOS susceptibility [**(A)**: allelic model, **(B)**: heterozygous model, **(C)**: homozygous model, **(D)**: recessive model, **(E)**: dominant model].

SUPPLEMENTARY FIGURE S6 Funnel plots of the five genetic models for the association between UCSNP-56 and PCOS susceptibility [**(A)**: allelic model, **(B)**: heterozygous model, **(C)**: homozygous model, **(D)**: recessive model, **(E)**: dominant model].

SUPPLEMENTARY FIGURE S7 Funnel plots of the five genetic models for the association between UCSNP-63 and PCOS susceptibility [**(A)**: allelic model, **(B)**: heterozygous model, **(C)**: homozygous model, **(D)**: recessive model, **(E)**: dominant model].

SUPPLEMENTARY FIGURE S8 TSA plot of the allelic model for the association between UCSNP-43 and PCOS susceptibility among mixed ethnicities.

SUPPLEMENTARY FIGURE S9 TSA plot of the dominant model for the association between UCSNP-43 and PCOS susceptibility among mixed ethnicities.

SUPPLEMENTARY FIGURE S10 TSA plot of the homozygous model for the association between UCSNP-44 and PCOS susceptibility among Asians.

SUPPLEMENTARY FIGURE S11 TSA plot of the recessive model for the association between UCSNP-44 and PCOS susceptibility among Asians.
