# Supplementary figures and images for: Association of Calpain10 polymorphisms with polycystic ovarian syndrome susceptibility: a systematic review and meta-analysis with trial sequential analysis
Source: Front Genet. 2023 Sep 1;14:1153960. doi: 10.3389/fgene.2023.1153960 (PMC10505618; doi:10.3389/fgene.2023.1153960)

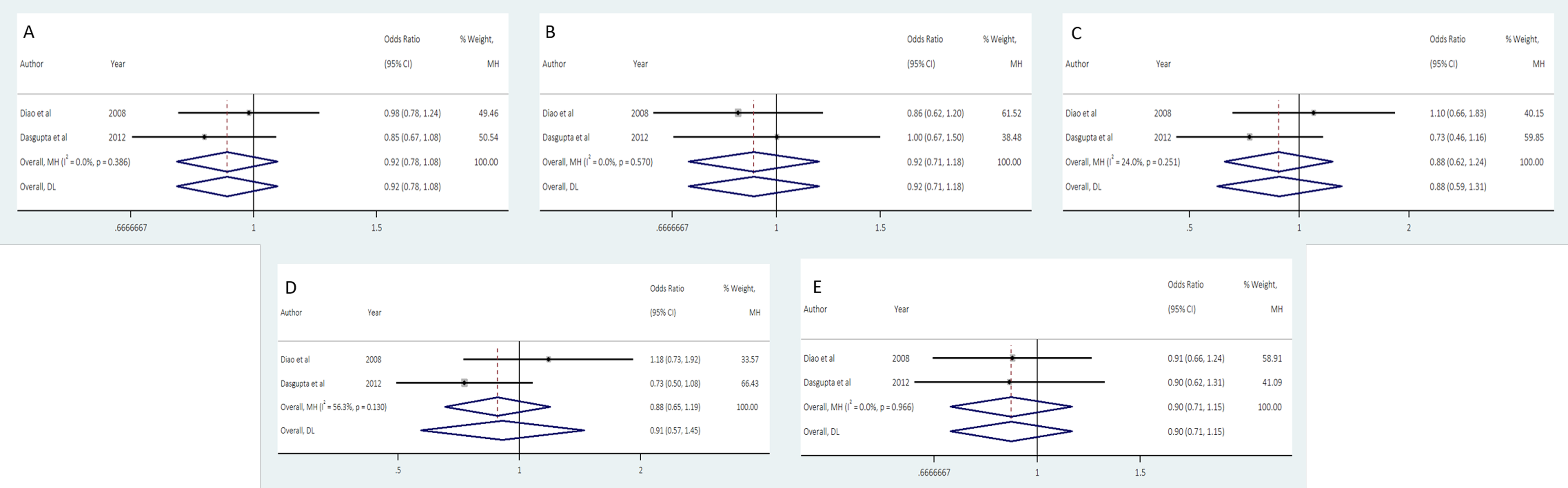

Supplement: Supplementary file 1 [file DataSheet1.ZIP › Supplementary Figure S1-S11/Figure S1.tif]

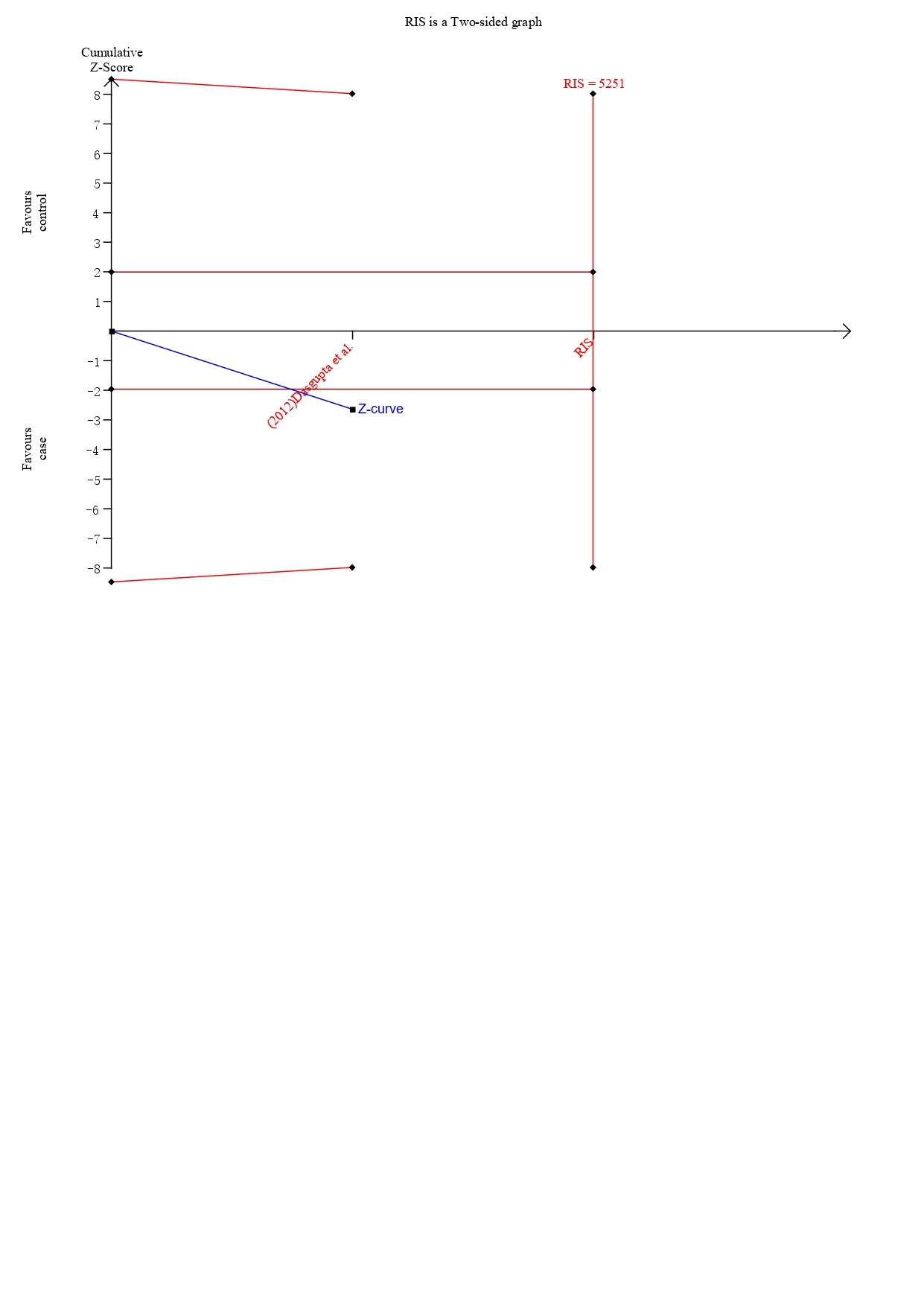

Supplement: Supplementary file 1 [file DataSheet1.ZIP › Supplementary Figure S1-S11/Figure S10.jpg]

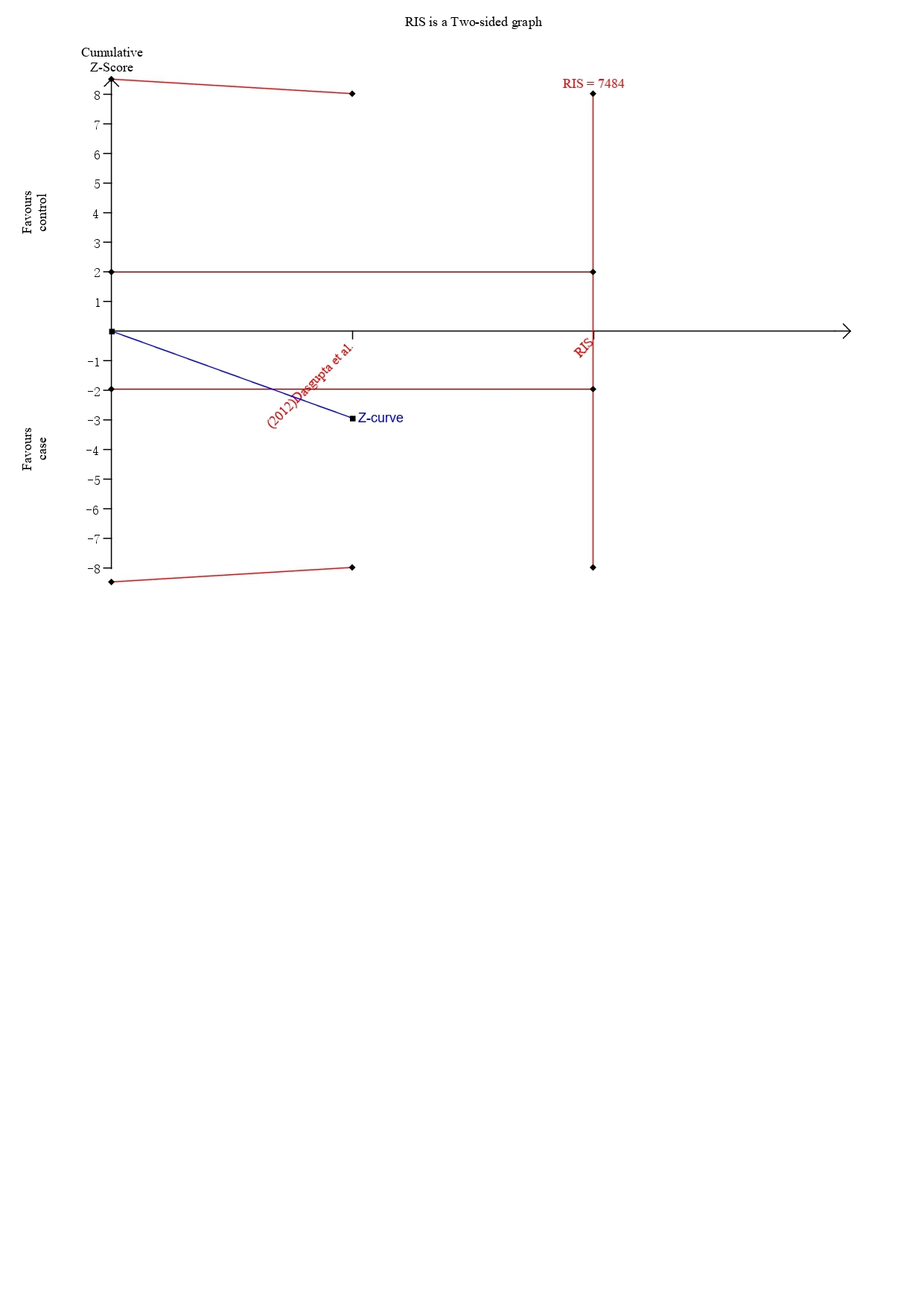

Supplement: Supplementary file 1 [file DataSheet1.ZIP › Supplementary Figure S1-S11/Figure S11.jpg]

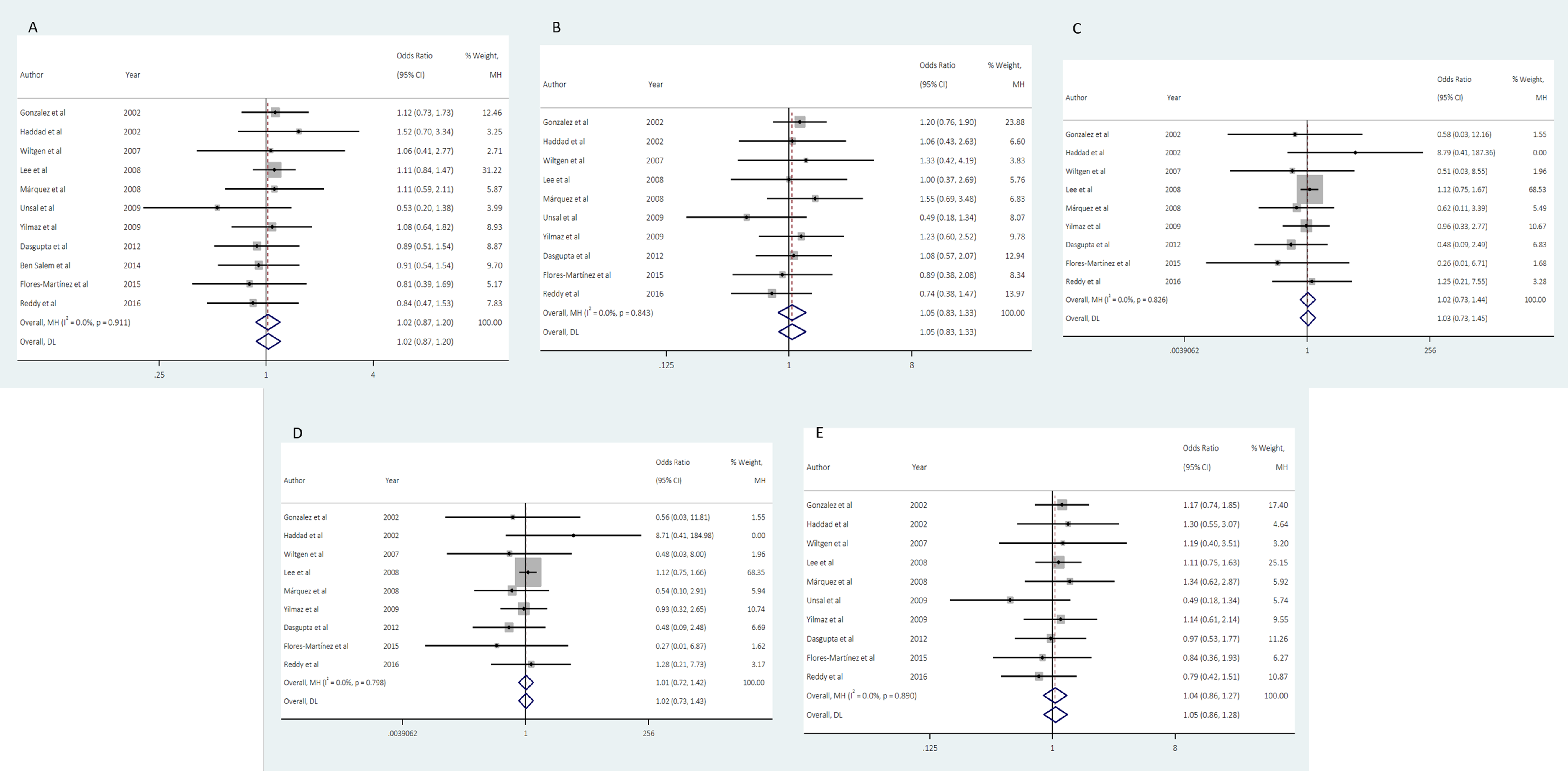

Supplement: Supplementary file 1 [file DataSheet1.ZIP › Supplementary Figure S1-S11/Figure S2.tif]

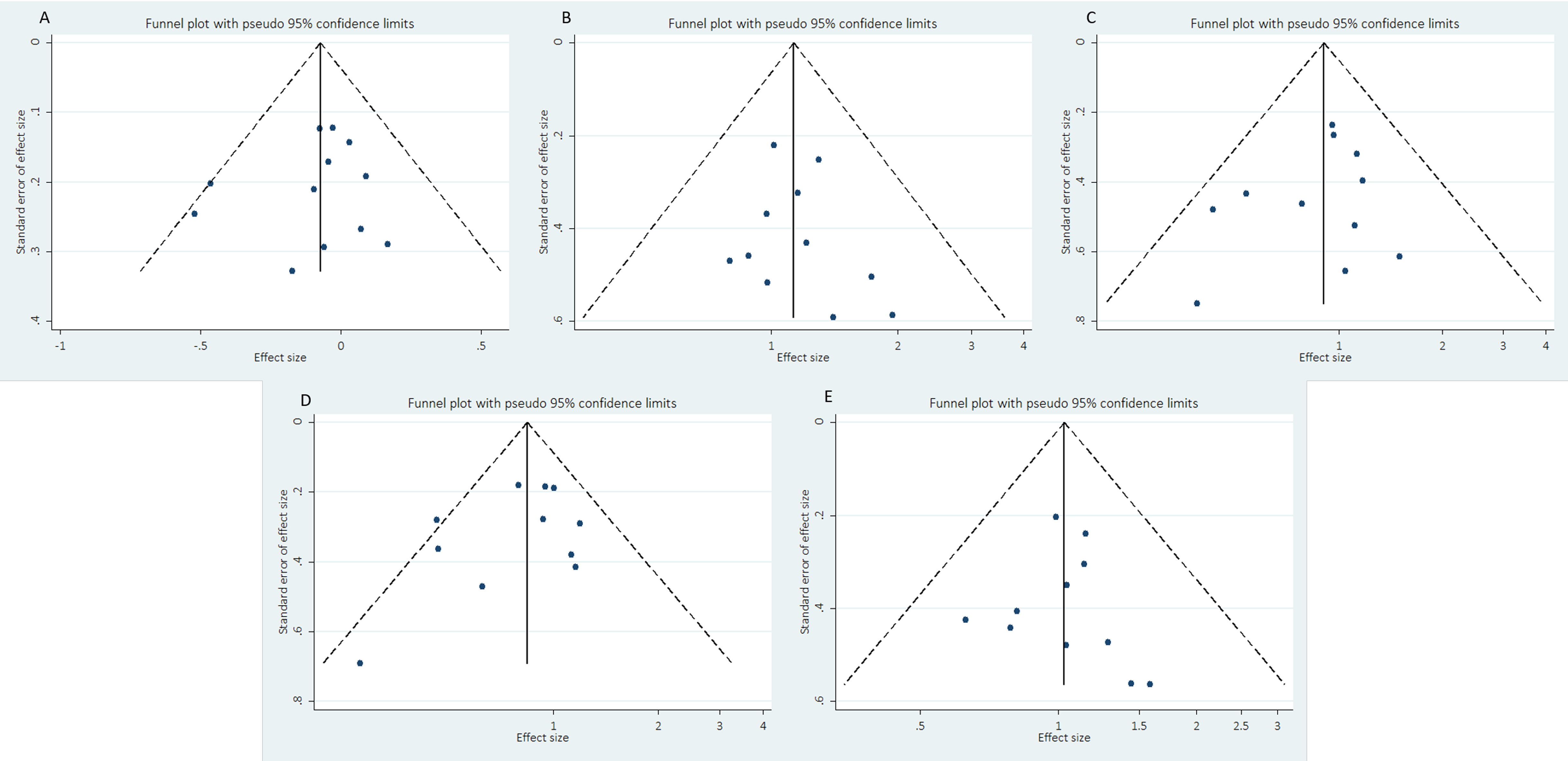

Supplement: Supplementary file 1 [file DataSheet1.ZIP › Supplementary Figure S1-S11/Figure S3.tif]

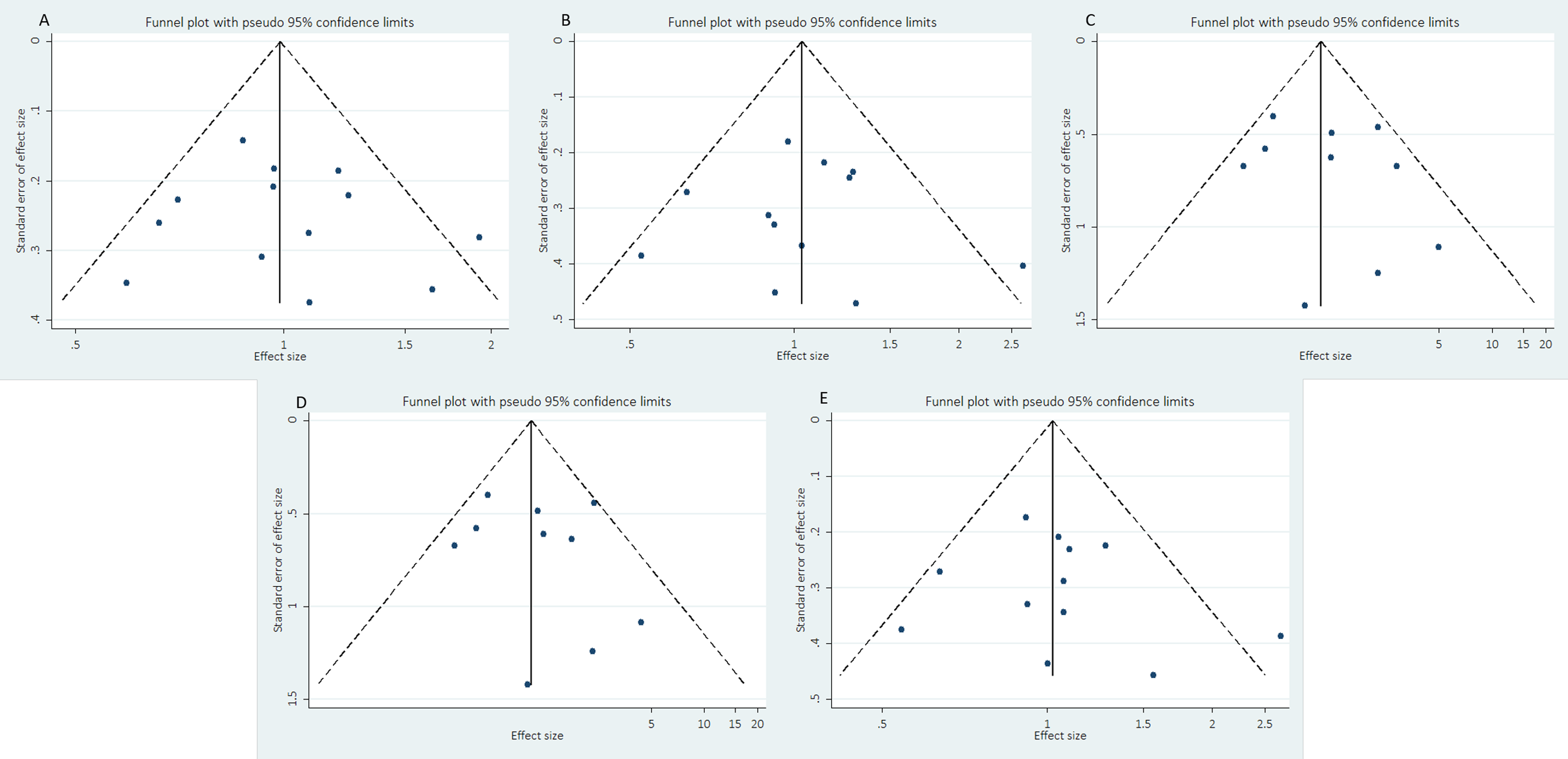

Supplement: Supplementary file 1 [file DataSheet1.ZIP › Supplementary Figure S1-S11/Figure S4.tif]

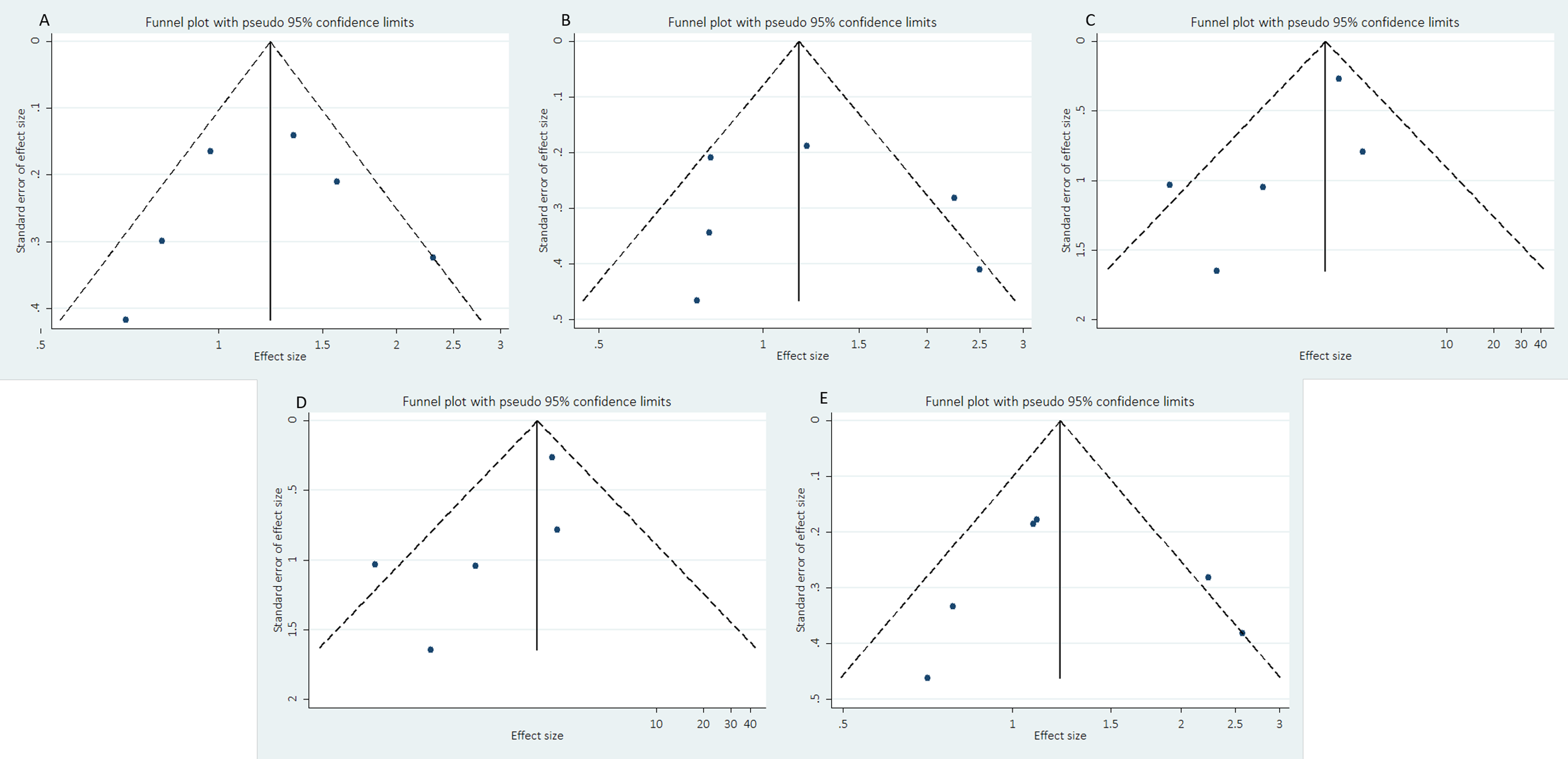

Supplement: Supplementary file 1 [file DataSheet1.ZIP › Supplementary Figure S1-S11/Figure S5.tif]

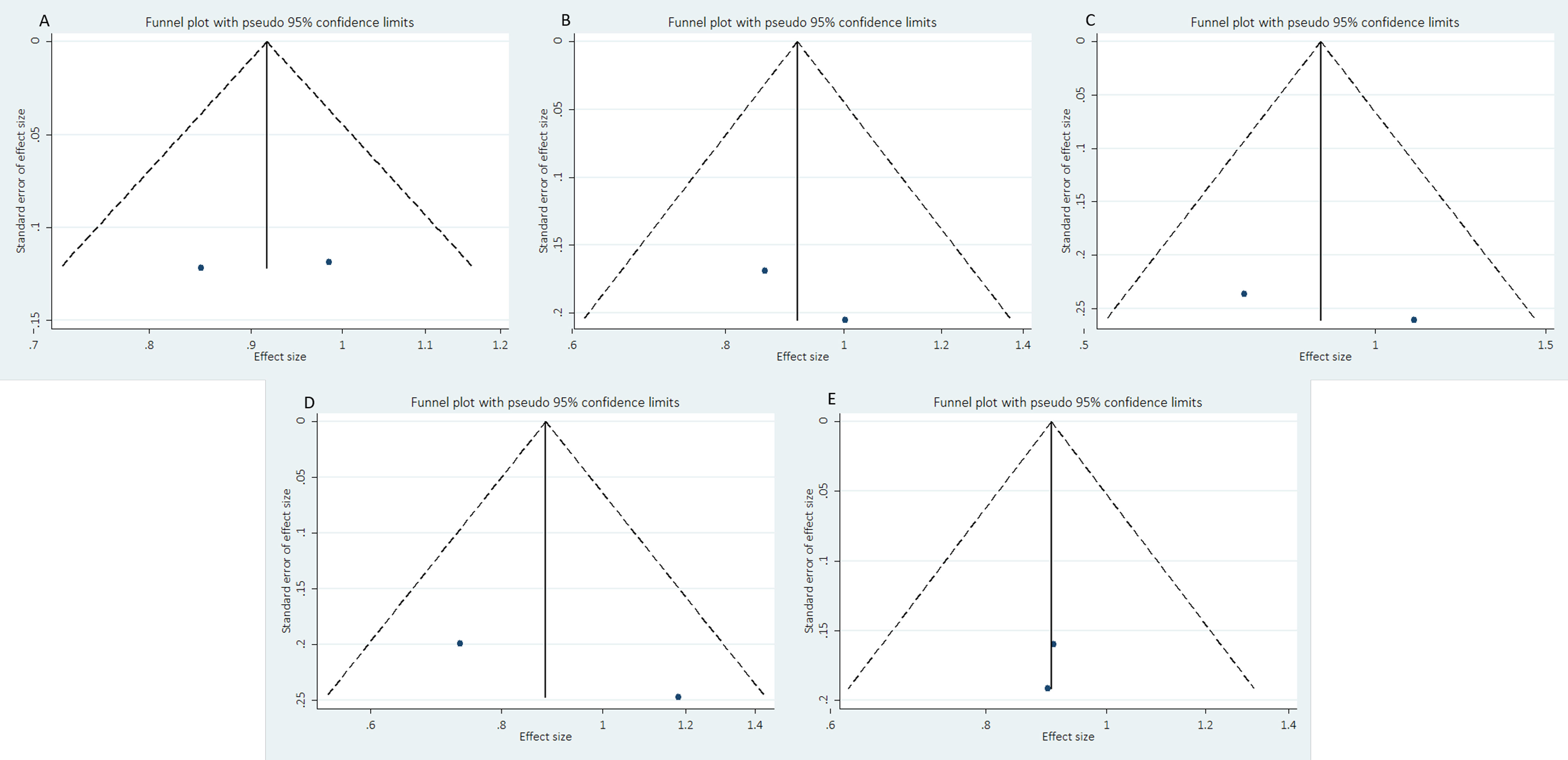

Supplement: Supplementary file 1 [file DataSheet1.ZIP › Supplementary Figure S1-S11/Figure S6.tif]

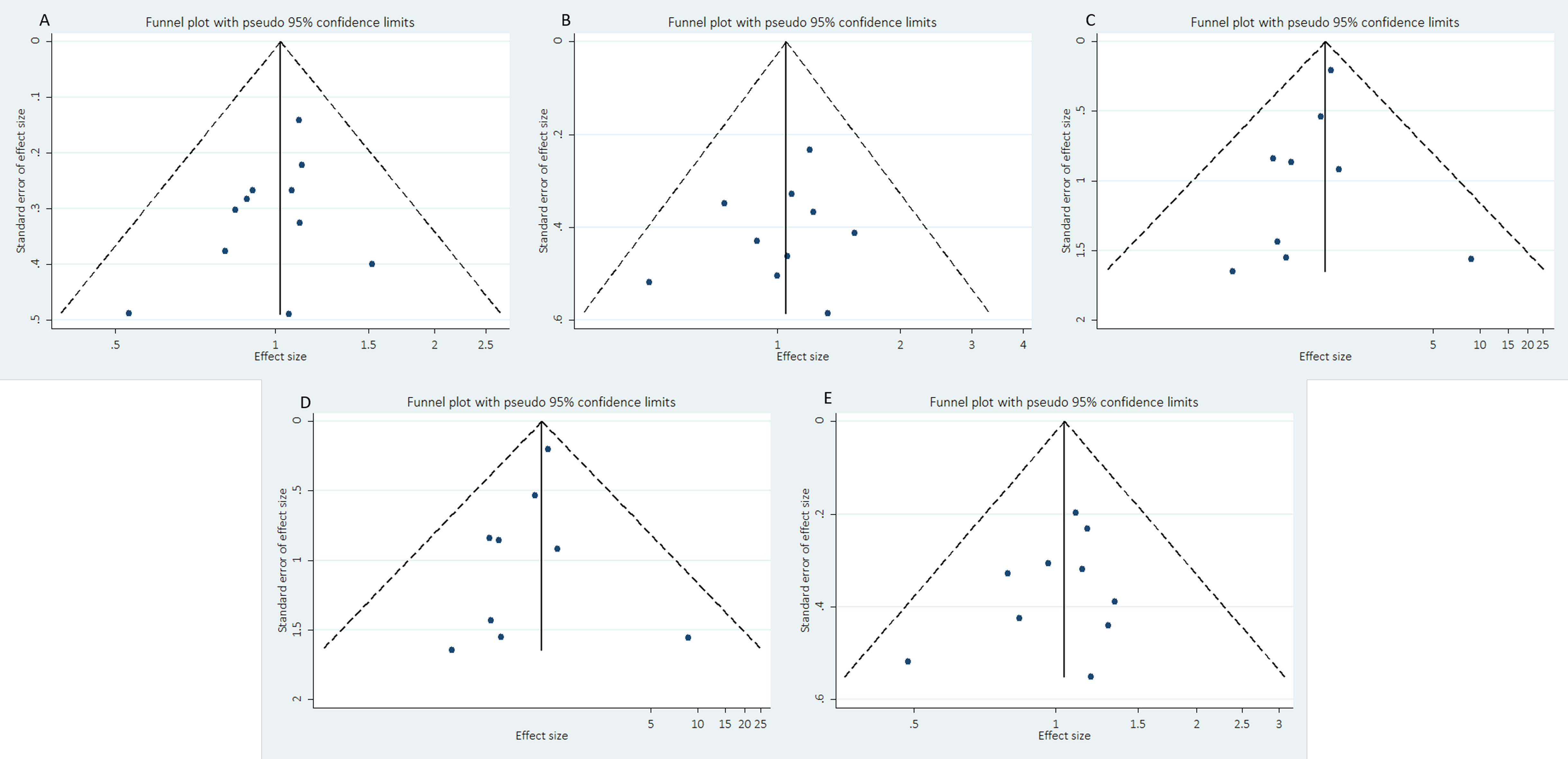

Supplement: Supplementary file 1 [file DataSheet1.ZIP › Supplementary Figure S1-S11/Figure S7.tif]

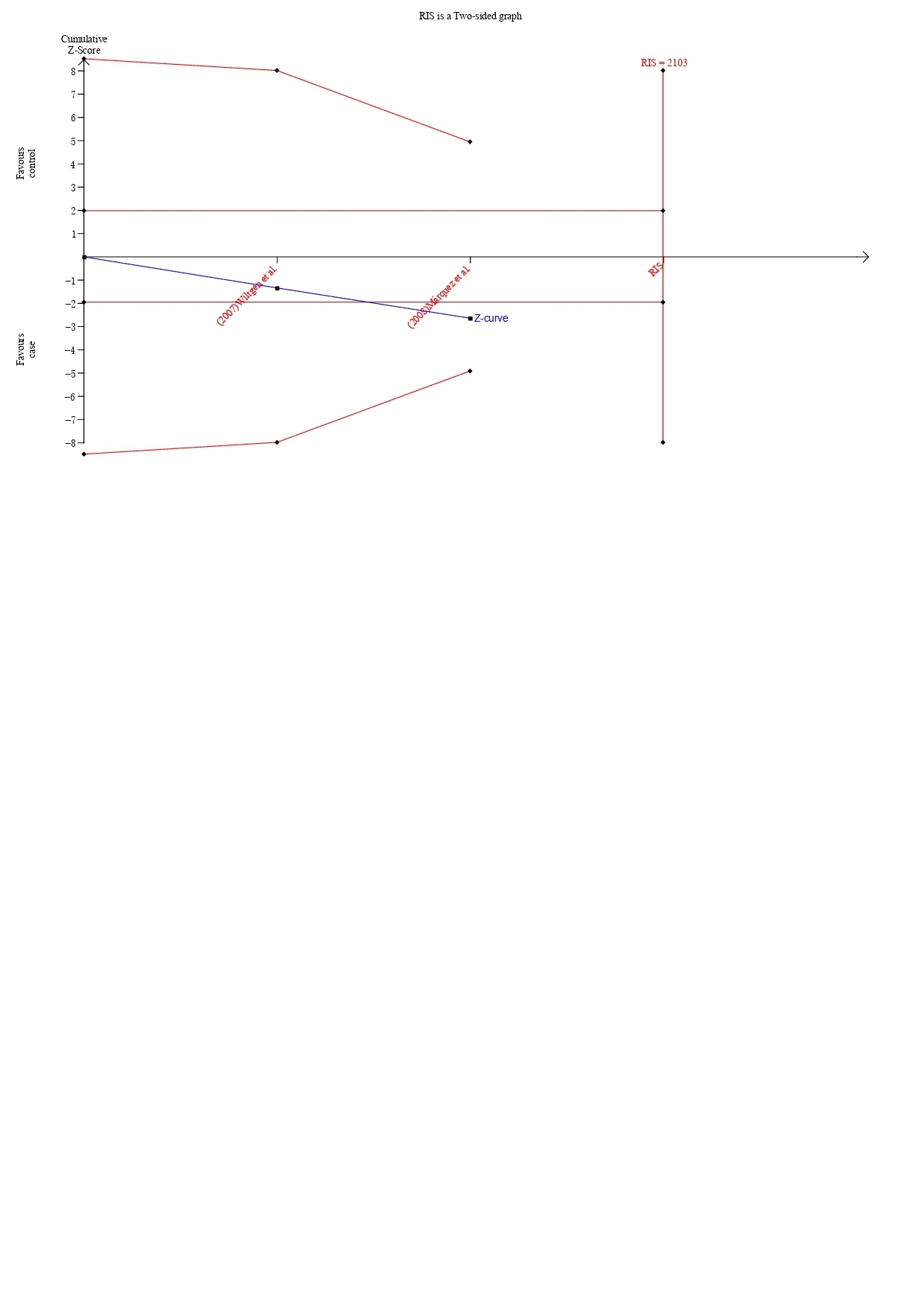

Supplement: Supplementary file 1 [file DataSheet1.ZIP › Supplementary Figure S1-S11/Figure S8.jpg]

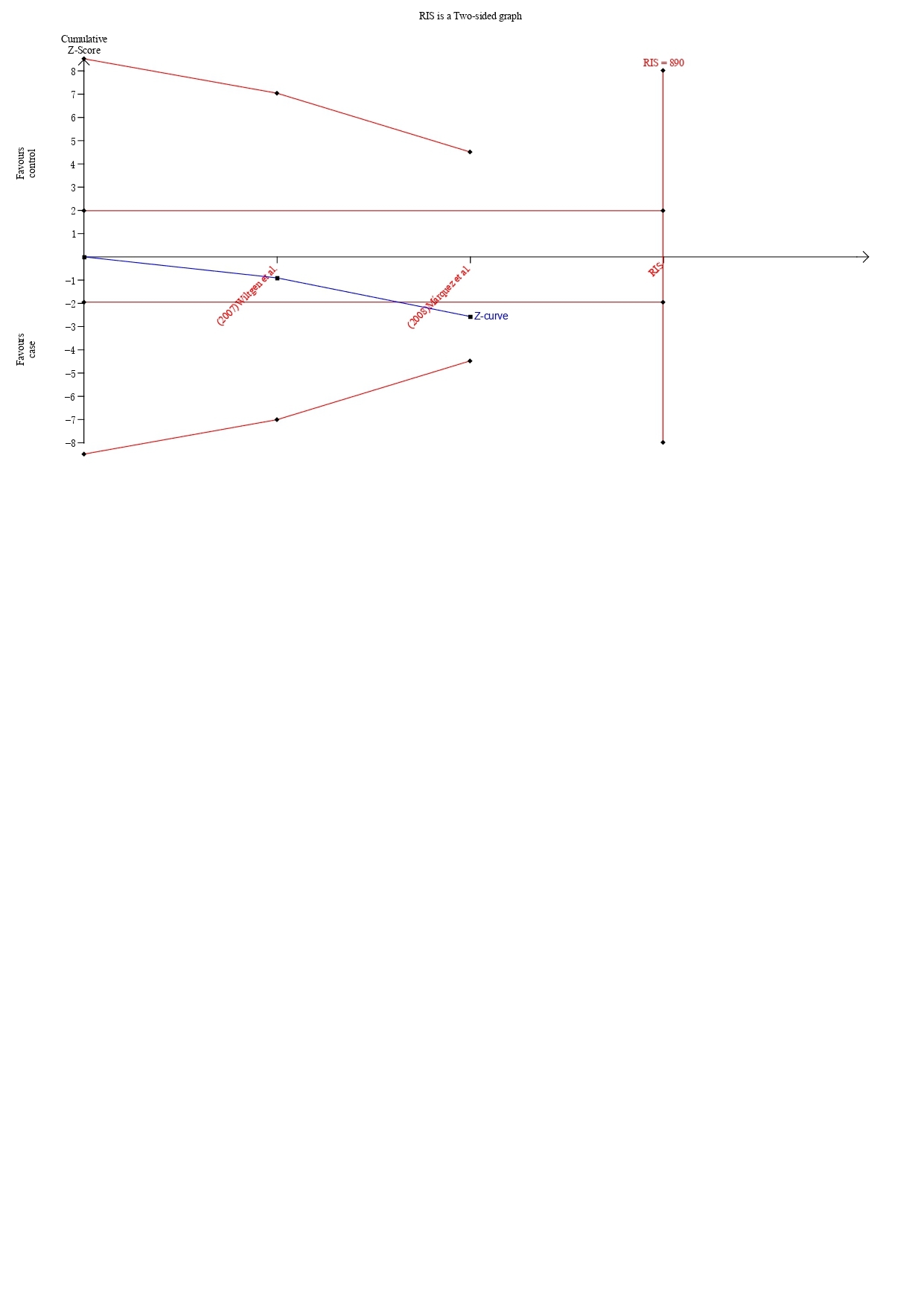

Supplement: Supplementary file 1 [file DataSheet1.ZIP › Supplementary Figure S1-S11/Figure S9.jpg]
